# A protocol for a randomised controlled, double-blind feasibility trial investigating fluoxetine treatment in improving memory and learning impairments in patients with mesial temporal lobe epilepsy: Fluoxetine, Learning and Memory in Epilepsy (FLAME trial)

**DOI:** 10.1186/s40814-019-0474-x

**Published:** 2019-07-06

**Authors:** Cheney J. G. Drew, Mark Postans, Cateno Petralia, Rachel McNamara, Philip Pallmann, Dave Gillespie, Lisa H. Evans, Nils Muhlert, Mia Winter, Khalid Hamandi, William P. Gray

**Affiliations:** 1 Centre for Trials Research, Cardiff University, Heath Park, Cardiff, CF14 4YS UK; 2 Cardiff University Brain Research Imaging Centre (CUBRIC), Maindy Road, Cardiff, CF24 4HQ UK; 3 School of Psychology, Cardiff University, Tower Building, 70 Park Place, Cardiff, CF10 3AT UK; 4 Division of Psychological Medicine and Clinical Neurosciences, University Hospital Wales, Cardiff and Vale University Health Board, Heath Park, Cardiff, CF14 4XW UK; 5 Division of Neuroscience and Experimental Psychology, Manchester University, Manchester, UK; 6 Department of Clinical Neuropsychology, University Hospital Wales, Cardiff, CF14 4XW UK; 7 The Alan Richens Welsh Epilepsy Centre, University Hospital Wales, Cardiff, CF144XW UK; 8 Neuroscience and Mental Health Research Institute, Hadyn Ellis Building, Maindy Road, Cardiff, CF24 4HQ UK

**Keywords:** Temporal lobe epilepsy, Hippocampal sclerosis, Fluoxetine, Allocentric learning

## Abstract

**Background:**

People with temporal lobe epilepsy (TLE) report significant problems with learning and memory. There are no effective therapies for combatting these problems in people with TLE, resulting in an unmet therapeutic need. The lack of treatment is, in part, due to a poor understanding of the neurobiology underlying these memory deficits. We know that hippocampal neurogenesis, a process believed to be important in learning and memory formation, is permanently reduced in chronic TLE, and this may go some way to explain the learning and memory impairments seen in people with TLE.

The common anti-depressant drug fluoxetine has been shown to stimulate neurogenesis both in the healthy brain and in neurological diseases where neurogenesis is impaired. In an animal model of TLE, administration of fluoxetine was found to restore neurogenesis and improve learning on a complex spatial navigational task. We now want to test this effect in humans by investigating whether administration of fluoxetine to people with TLE can improve learning and memory.

**Methods:**

This is a single-centre randomised controlled, double-blind feasibility trial. We plan to recruit 20 participants with a diagnosis of TLE and uni-lateral hippocampal sclerosis, confirmed by 3T MRI. Eligible participants will undergo baseline assessments of learning and memory prior to being randomised to either 20 mg/day fluoxetine or matching placebo for 60 days. Follow-up assessments will be conducted after 60 days of trial medication and then again at 60 days after cessation of trial medication. Feasibility will be assessed on measures of recruitment, retention and adherence against pre-determined criteria.

**Discussion:**

This trial is designed to determine the feasibility of conducting a double-blind randomised controlled trial of fluoxetine for the treatment of learning and memory impairments in people with TLE. Data collected in this trial will inform the design and utility of any future efficacy trial involving fluoxetine for the treatment of learning and memory in people with TLE.

**Trial registration:**

EudraCT 2014-005088-34, registered on May 18, 2015

## Backgrounds

Epilepsy is the most common chronic neurological disorder affecting between 4 and 7 in 1000 people in developed countries [1]. Temporal lobe epilepsy (TLE) is the most common form of drug refractory focal epilepsy [2]. It is thought that TLE accounts for up to 40% of all focal epilepsy diagnoses [3]. In TLE, seizures arise from a focal point within the temporal lobe and can manifest with a range of seizure types including simple partial seizures (typically producing an aura or strong emotional (fear) or physiological (smell) responses), complex partial seizures (consciousness is altered and repeated movements such as grabbing at clothes or lip smacking are observed) or secondary generalised tonic-clonic seizures.

In addition to seizures, people with TLE also experience a number of neuropsychological co-morbidities with memory dysfunction reported as the most common neuropsychological effect of TLE [4–6]. Over half of patients with epilepsy rate their memory problems as moderate to severe [7], contributing significantly to their adverse quality of life [8] and impacting considerably on their daily functioning. At present, there are no pharmacological or non-pharmacological strategies available to try and combat the learning and memory problems associated with TLE. Indeed, although the neuropsychological problems associated with chronic epilepsy and TLE in particular are well documented, there is a dearth of research into combatting this common issue, thus highlighting cognitive dysfunction in TLE as a significant unmet therapeutic need.

In general terms, the cause of epilepsy is poorly understood and cannot be defined for all epilepsy patients. However, TLE is strongly linked with hippocampal sclerosis (HS), which describes a general atrophy and scarring of the hippocampus. This can be either on one (unilateral) or both (bilateral) sides of the brain [9].

The hippocampus is a brain structure known to be important in all stages of episodic and spatial memory processing including encoding, consolidation and retrieval [10–12]. Spatial learning, to locate a specific target or goal, is known to employ different strategies as follows: allocentric learning describes the process by which a person creates a cognitive map by remembering the specific spatial relationship between the surrounding environment and the target, and egocentric learning describes how a person will learn the spatial relationship between the goal and their own body. Both rodent [13] and human studies [14] using the Morris Water Maze paradigm and functional magnetic resonance imaging (MRI) [15, 16] have demonstrated that the hippocampus is necessary for allocentric but not egocentric or cued learning. This may indicate why patients with TLE have such problems with spatial learning and memory recall. Further, we have recently demonstrated that patients with unilateral HS have significantly less efficient allocentric learning than healthy controls [17] and although this can be overcome by increased training, they also forget the learned task more quickly.

The hippocampus is a site for adult neurogenesis [18], the formation of new brain cells in the developed brain. Although in the past the role of neurogenesis in spatial learning has been debated [19], paradigms which require a higher cognitive load (such as allocentric learning) have identified a significant role for neurogenesis in both the acquisition [20] and retrieval [21] of spatial memories. Further, neurogenesis appears to be particularly important for supporting pattern separation of similar stimuli [22], which supports allocentric learning.

In animal models status epilepticus permanently alters hippocampal neurogenesis [23–25]. Not only does status epilepticus alter the amount of newly formed cells [26] but also reduces the resultant connectivity of newly born neurons [27, 28]. Deficits in neurogenesis have been confirmed in patients with TLE [29] which is associated with decreased performance in memory and learning tasks [30]. Thus, impaired neurogenesis may be one possible biological mechanism that can account for the manifestation of memory and learning deficits in patients with TLE. We have validated the kainate model of mesial TLE in rats, by identifying reduced neurogenesis and an exactly matching pattern of spatial learning deficits to that seen in patients with hippocampal sclerosis (virtual water maze) [17, 31].

Rates of neurogenesis can be affected by a number of different factors including depression [32], stress[33], exercise [34] and neurotrophic factors [35, 36]. It has now been established that pharmacological agents, particularly anti-depressants such as fluoxetine can increase levels of neurogenesis in rats and primates [37]. Critically, it has been demonstrated that learning impairments and altered neurogenesis exhibited in the rat kainate model of TLE can be effectively restored by fluoxetine treatment [31].

The currently available research evidence strongly suggests that impairment in hippocampal neurogenesis underlies the deficits in allocentric learning seen in patients with TLE. Further, evidence from animal models of TLE shows that fluoxetine is effective in improving neurogenesis sensitive learning [31], which leads to the question, can fluoxetine treatment enhance adult human neurogenesis and thus improve learning and memory deficits in people with TLE and HS?

Fluoxetine is a relatively cheap, widely available and generally well-tolerated medication with a good safety profile, and is widely prescribed in people with epilepsy [38], owing to the high comorbidity of depression and epilepsy [39]. Whilst the British National Formulary (BNF) urges caution for the prescription of fluoxetine in this population based on experimental increased seizure risk [40], most studies suggest the anti-depressants do not worsen seizures and may have a protective, anti-convulsant effect at therapeutic doses [41–46]. Further, there is evidence to suggest that fluoxetine can increase suicidal thoughts and suicidal ideation in adolescents [47–49], but this is only in people being treated for active ongoing depression. Whilst these factors pose a potential risk, it is possible to mitigate these risks, and thus, fluoxetine treatment in the context of remedying an unmet therapeutic need in people with TLE warrants further investigation.

Learning and memory in people with TLE can be measured using a number of hippocampal-dependent measures of learning. This includes the virtual water maze (a direct correlate of the widely used rodent version and described in [17]), pattern separation tasks and SenseCam paradigm. Pattern separation tasks serve as a highly hippocampal-dependent test of location memory that can be automated in both rodent [50] and human [51] models of testing. There is evidence to suggest that discrimination of pattern separation, especially when finely spaced, is dependent on neurogenesis [52]. The SenseCam paradigm allows the testing of learning and memory in a real-world setting which may be more applicable to situations of daily living where memory deficits may be more problematic. This has previously been used to demonstrate accelerated forgetting in people with transient epileptic amnesia [53].

Here, we describe a feasibility trial to see if the investigation of the beneficial effects of fluoxetine treatment in animal models of epilepsy can be successfully translated into the human epilepsy population, to assess its potential as an effective therapy for the memory and learning impairments associated with TLE. This feasibility trial will allow us to examine a number of trial processes such as recruitment, retention and adherence of trial participants and to calculate potential effect sizes on outcome assessments for designing an appropriately powered phase II efficacy trial to determine if fluoxetine has any beneficial effects on the learning and memory of people with TLE.

## Methods

### Trial design and setting

**Fig. 1:**
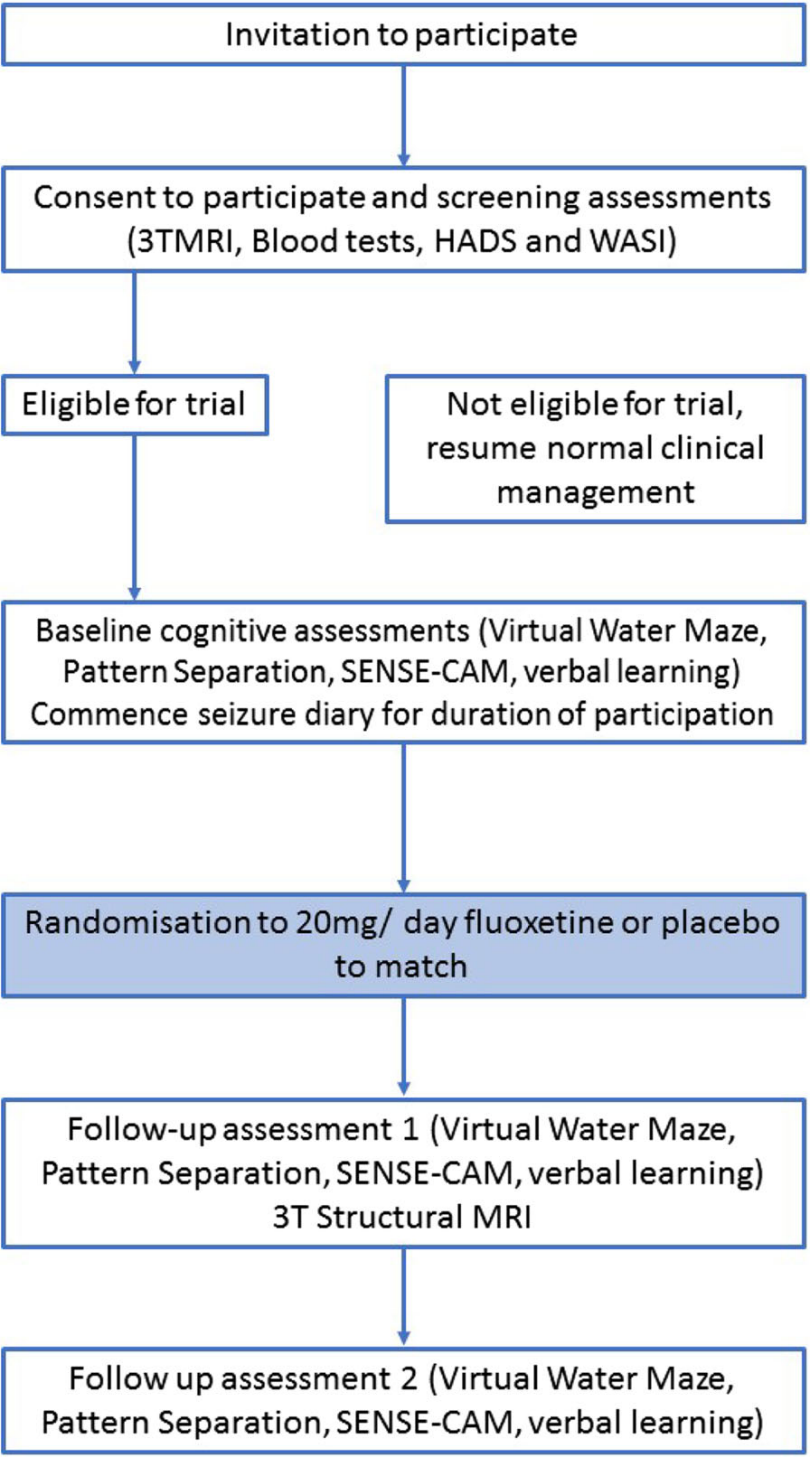
Schematic of trial design

FLAME is a single-centre, double-blind, randomised placebo-controlled feasibility trial of the effect of fluoxetine on learning and memory deficits in patients with TLE. All research activities will be conducted between University Hospital Wales, Cardiff, and clinical research facilities within Cardiff University. Eligible participants will perform baseline cognitive assessments prior to randomisation to trial treatment or placebo. Following randomisation on the last day of the baseline assessments, participants will commence study treatment and after a treatment period of 60 days, the cognitive assessments will be repeated whilst continuing the trial medication. Participants will cease trial medication at the end of follow-up assessment one and will begin a washout period of 60 days, after which they will complete the final battery of cognitive assessments at follow-up assessment 2 (Fig. 1).

### Primary objective

The primary objective of this trial is to determine the feasibility of conducting a double-blind, randomised controlled trial of the effect of fluoxetine on learning and memory in people with TLE.

### Secondary objectives

Secondary objectives of this trial are largely exploratory and are to (1) determine the effect of 60 days of oral fluoxetine treatment on spatial and verbal learning in people with TLE and any ongoing effects after a further 60 days of treatment withdrawal, (2) determine if people with TLE differ in the severity of their learning deficit dependent on which side hippocampal sclerosis occurs, (3) determine if people with TLE show a deficit in pattern separation and if these deficits can predict a response to fluoxetine, (4) investigate if hippocampal microstructure correlates with allocentric learning and memory deficits and/or the response to fluoxetine and (5) determine if fluoxetine alters the seizure frequency of people with TLE.

### Recruitment and consent

We aim to recruit 20 participants for this trial; ideally 10 people with right-handed HS and 10 people with left-handed HS. The sample size was set at the maximum that was deemed achievable within the timeframe and budget of the study and given the size of the target population and level of engagement required. It will allow us to estimate the recruitment, retention and adherence rates with 95% confidence intervals of no more ± 22 percentage points. Potentially eligible participants will be identified from epilepsy clinic lists within the Alan Richen’s Epilepsy Unit in University Hospital Wales, Cardiff, and associated clinical databases. Additional participant identification centres (PICs) will be used across South Wales, Bristol and Southampton. Potentially eligible participants will either be sent an invitation letter with the participant information sheet (PIS) or will be handed the PIS during their routine clinic visit. Along with the PIS, participants will also be given a contact form on which they can indicate their interest, in participating in the trial along with contact details enabling a researcher to contact them. The trial will also be externally advertised on third sector websites such as Epilepsy Action to allow for participant self-referral.

Those participants that return the contact form or otherwise indicate their willingness to participate will be asked to provide written consent. Consent will be taken by trained and qualified trial researchers.

Additionally, we will seek to recruit a family member or care giver for each participant recruited who can accompany them to assessments. These additional recruits will act as controls for one of the cognitive assessments employed (SenseCam). Full written consent will be also obtained from healthy controls prior to their inclusion in the trial.

### Inclusion/exclusion criteria

The inclusion/exclusion criteria have been selected on the basis of what we believe would be required for an efficacy trial. To ensure eligibility for study recruitment, participants must fulfil all the inclusion criteria as follows: (1) aged between 18 and 65 years, (2) confirmed clinical diagnosis of TLE, (3) unilateral HS confirmed by 3T MRI (to enable investigation of potential differences between right- and left-handed HS) and (4) prepared to take adequate contraception for the duration of the trial. Participants will be excluded if they meet any of the exclusion criteria as follows: (1) bi-lateral HS; (2) presence of significant active anxiety or depression (indicated by a score of 11 or more on the Hospital Anxiety and Depression Scale (HADS) [54]) because an ongoing depressive or anxious state may affect performance on the behavioural tests included in this study, which could lead to difficulties in determining whether any change in outcome measures following fluoxetine treatment, should they occur, were due to treatment of depression/anxiety or the effects of fluoxetine on hippocampal neurogenesis; (3) current treatment with a selective serotonin re-uptake inhibitor (SSRI), as participants would already be receiving the intervention (4) lacking capacity or an Intelligence Quotient (IQ) of less than 75 (assessed by the Wechsler Abbreviated Scale of Intelligence (WASI-II) [55]); and (5) presence of poorly controlled seizures in participants undergoing active anti-epileptic drug (AED) changes, as it known that ongoing seizures may affect cognition directly.

The remaining exclusion criteria are included for safety purposes of study assessments or prescription of fluoxetine: (6) not suitable for MRI; (7) pregnancy or breastfeeding; (8) participation in another clinical trial of an investigational medicinal product; (9) previous adverse reaction to fluoxetine; (10) taking any contraindicated medication detailed in the Summary of Product Characteristics (SPC) (such as monoamine oxidase inhibitors); (11) hepatic impairment defined by liver enzymes elevated to 2.5 times the upper limit of the normal range; (12) congenital long QT syndrome or any family history of any clinical condition predisposing to arrhythmia; (13) taking tamoxifen; and (14) taking St. John’s wort (as this also alters neurogenesis and the behavioural responses to chronic stress [56]).

Participants with a diagnosis of TLE as suggested by electroencephalography (EEG) recording without confirmation of HS on MRI will not be included as epileptiform discharges seen on EEG commensurate with a diagnosis of TLE cannot be localised to the mesial temporal lobe with a high degree of accuracy and may originate in the neocortex. As the rationale for this study is intrinsically linked to potential deficits of neurogenesis in the hippocampus, participants without confirmation of HS on MRI will be excluded. This also applies to participants who may have bi-lateral EEG signs of TLE but only uni-lateral HS on MRI.

### Screening assessments

Potentially eligible participants will be invited to attend a screening assessment. This will include a medical history, blood tests for liver enzymes and electrolytes, completion of the HADS, completion of the WASI and a structural 3T MRI to confirm laterality of HS (see Table 1). Any participant who declares a history or family history of heart conditions predisposing to arrhythmia will also receive an electrocardiogram (ECG). Participants who are found to have any of the following, bi-lateral HS, an intelligence quotient (IQ) of 74 or below, a HADS score of 12 or more, an aspartate transferase level more than 2.5 times the upper limit of normal, hypokalemia, hypomagnesia or an abnormal ECG (Q-T interval prolongation, ventricular arrhythmia or Torsades de pointes), will be excluded from the trial. As the HADS is a guideline rather than absolute measure of anxiety or depression, participants who score 11 (borderline) will undergo review by a consultant psychiatrist. If the psychiatrist does not think that the participant has active ongoing anxiety or depression, they will be able to continue in the trial.

**Table 1:** Schedule of trial assessments

Procedure	Schedule											
	Eligibility assessment	Baseline assessments			Study drug	Follow-up assessment 1				Follow up assessment 2		
Visit number	1	2	3	4		5	6	7	8	9	10	11
Time-point of visit	T-1 to 4 weeks	T0	T1	T-21	T22-T104	T83	T84	T[97-104]	T104	T164	T165	T185
Written informed consent	X											
HADS	X											
WASI	X											
Blood tests (liver function, electrolytes)	X											
ECG (where indicated)	X											
MRI Safety Questionnaire	X							X				
Issue of seizure diary	X											
Structural MRI	X							X				
Review of seizure frequency		X	X	X		X	X		X	X	X	X
QOLIE-31		X				X				X		
Written Cognitive Assessments		X										
Virtual Water Maze Task		X	X	X		X	X		X	X	X	X
Pattern separation task		X				X				X		
Word Lists		X	X	X		X	X		X	X	X	X
Medical Outcomes Sleep Survey		X				X				X		
Issue of SenseCam		X				X				X		
Testing of SenseCam		X	X	X		X	X		X	X	X	X

### Trial assessments

Participants will be asked to perform a number of written cognitive tests at baseline only; the purpose of which is to compare participant performance in novel tests against standard tests of cognition at baseline and identify potential floor or ceiling effects. These include the Test of Pre-Morbid Functioning (TOPF) [57], the Brain Injury Rehabilitation Trust Memory and Information Processing Battery (BMIPB) [58] and the Everyday Memory Questionnaire (EMQ) [59]. Participants will also be asked to complete the Chalfont Seizure Severity Scale [60].

For testing time-point (baseline, follow-up one and follow-up two), participants will be asked to perform a series of memory and learning tasks (Table 1). Additionally, they will be asked to complete the shorter version of the Quality of Life in Epilepsy Questionnaire (QOLIE-31) [61] and the Medical Outcomes Sleep Survey [62] to assess whether any observed alterations in sleep quality relating to fluoxetine mediate any potential effects on learning and memory. The cognitive tests include the virtual water maze [17, 31], a pattern separation task [51], SENSE-CAM task [53] and verbal learning and memory testing using word lists.

The virtual water maze is a computer generated, the four-walled room containing a pool which can be navigated by the participant using the arrow keys on a keyboard [17, 31]. An escape platform is located within the pool and participants must navigate to this escape platform. Participants will be given 50 trials to practise navigating the arena. Participants will receive two sessions; in the first, they will learn the cued navigation and allocentric learning tasks (testing day 1 only) and the second session will be a probe trial to test the participant’s learning. Session 2 will be repeated on days 1, 2 and 21 of each testing block. In the initial cued navigation task, participants must navigate to a visible platform within the arena from a wall facing starting position. If they do not locate the platform within 45 s, the researcher will demonstrate how to reach the platform. This is repeated for a total of six times, after which, the platform is removed and participants are asked to navigate towards where they think the platform is (they are told it is now invisible). The participant’s navigational pathway will be analysed to determine the amount of time spent in each quadrant, the latency and distance travelled to cross the platform area and the number of times they cross the platform location. In the allocentric task, participants are placed in the same arena, but this time with external wall cues. Participants are asked to navigate to an invisible escape platform in the pool within 45 s. Once this is achieved, they receive an on-screen alert to tell them they have successfully found the location of the platform and are given 5 s to view their position in relation to the external cues. This trial is repeated 20 times with the location of the platform remaining constant throughout but with the start position varied. At the end of the training, participants are subjected to a probe trial where the platform has been removed without their knowledge. The participant’s movements are traced for 45 s to record measures of navigation as in the cued navigation task. Following the probe trial, participants are given three more training trials as before to lessen the impact of the probe trial on their memory of the platform location. The second session is designed as a test of accelerated forgetting. Participants will be asked to navigate to the invisible platform, located in the same position as in session 1. The first trial will be a probe trial, with the platform removed unbeknownst to the participant and will last for 45 s. This will then be followed by three further training trials to minimise confusion from the preceding probe trial.

For the pattern separation task, participants enter a computer-generated arena facing a particular direction and are then presented with a visual stimulus located in one of 20 pre-determined spatial locations within the arena. The participant must then navigate to the stimulus using the arrow keys on a keyboard, and once at the stimulus, they must press the space bar to initiate the next part of the trial. For the choice phase, the participant is presented with four visual stimuli in a square formation; one corner is in the same spatial location as the previous stimulus and the other three stimuli are foils. The participant must navigate to the stimulus presented in the location of the original stimulus using extra-arena cues and press the space bar to indicate their choice within the 45 s time limit of the trial. Across trials, three separation distances between the grouped stimuli will be used (minimum, intermediate and maximum) in a pseudorandom order. Twenty-one test pairs (seven per condition) will be presented in total.

In the SenseCam task, participants will be asked to attend a local tourist attraction, accompanied by a family member or friend, who has given their consent to participate, whilst wearing a SenseCam camera which hangs around the neck at chest level. Following the excursion, the researcher will extract 4–5 snapshot images of six events in order to test the participant’s recall of the excursion. The person accompanying the participant will be presented with the same images and asked to recall details of the excursion in the same manner to provide control responses to those of the participant. Images will be presented on a laptop screen and participant will be given as long as they like to look at the image. They will be asked to recall the event pictured and secondary details associated with it—the events occurring either immediately before or after the presented event. Each correct recollection is given a one-point score giving a maximum possible score of 3 per picture presented. Participants will be tested on their recall on day 1, day 2 and day 21 of each testing block.

Verbal learning and memory will be tested with simple word lists generated from the MRC Psycholinguistics Database with the following word characteristics: letters = 4–6, concreteness = high [500–700], written frequency = low [1–50] (see [63]). Participants will be given a list of 20 words and they will be allowed to learn the words until they can accurately recall 80% of the list provided. Participant’s recall will then be re-tested after a 30-min delay on day 1, a 24-h delay on day 2 of the testing block and after a 3-week delay on day 21 of the testing block. On day 21, participants will also be assessed for residual memory through a recognition test of the original word list. They will be presented with a set of words containing some of the original words on the learned list (targets) and novel words (foils) in equal numbers and asked to identify the target words. For words that the participant identifies as one of the target words, they will then be asked to indicate whether (1) they could recollect any details about the presentation of the word (“Remember” responses), or (2) they judged the word to have been a target but could not recollect any details about its presentation (“Know” responses). The proportion of Remember and Know responses obtained through this Remember/Know procedure will then be used to derive estimates of recollection and familiarity [64, 65].

A 3T MRI will be performed at the screening assessment and will be repeated following trial treatment. The scanning protocol will include structural sequences required to confirm unilateral sclerosis (FLAIR as well as T1- and T2-weighted images), as well as a multi-shell diffusion-weighted sequence with which we will be able to derive voxel-wise maps of water diffusion indices. These will include diffusion tensor MRI (DT-MRI) measures such as fractional anisotropy (FA) and mean diffusivity (MD), which are sensitive to grey and white matter microstructural abnormalities in a variety of neurological conditions including temporal lobe epilepsy [66–68]. These measures can be extracted from specific regions-of-interest (ROIs) and compared pre- versus post-treatment to test whether fluoxetine treatment affects grey and/or white matter tissue microstructure (e.g., in the hippocampus and its associated white matter pathways). With these measures, we can also examine whether performance in the cognitive tasks is associated with DT-MRI measures in the same ROIs. Prior to receiving an MRI, participants will be required to complete a questionnaire to ensure that it is safe for them to have the scan.

All participants will be provided with seizure diaries at the start of the baseline assessments. They will be asked to record any seizures in the diary until they complete the final follow-up assessment. This diary will be reviewed at each follow-up and testing session to ensure that seizure frequency has not increased significantly.

### Randomisation, allocation concealment and blinding

Eligible participants will be randomised in a 1: 1 ratio (fluoxetine to placebo) using random permuted blocks, stratified by status of laterality of HS (left versus right) following baseline assessments. Each block of allocations will be equally balanced between active and placebo. A computer-generated sequence will select which permutation to use for every block of participants. Block size and permutations will remain concealed to the trial team. The trial treatment will be dispensed by the local clinical trial pharmacy according to the pre-specified list provided by the trial statisticians. Identical capsules containing either fluoxetine or placebo will be packaged in identical high-density polyethlyene containers with tamper evident closure. Each package will be labelled in compliance with Annex 13 and according to EU mandated Good Manufacturing Practice. A detachable tear off section will allow maintenance of blinding at the point of dispensation. Details of allocation will be kept within the participant accountability records within pharmacy and will not be revealed to the trial team unless emergency unblinding is required. Unblinding will be permitted if the participant (1) withdraws due to a significant decrease in mood, (2) exhibits an episode of status epilepticus, (3) experiences severe withdrawal symptoms and (4) requires the prescription of any medication that should not be taken in conjunction with fluoxetine as detailed in prescribing guidelines. If any of these criteria are met, the information will be passed to the chief investigator (CI) or clinically qualified delegate who will confirm the need for unblinding. The CI or trial manager will contact the dispensing pharmacy to review the accountability records for that participant. In emergency situations, the CI or clinically qualified delegate will contact the on-call pharmacist to review the relevant participant accountability log.

### Trial treatment

Following randomisation, participants will be allocated to receive either 20 mg fluoxetine per day or matched placebo. Fluoxetine will be obtained from Bristol Laboratories and over encapsulated at St. Mary’s Pharmaceutical Unit, Cardiff. Placebo to match will be manufactured in identical capsules using maize-based cellulose as the capsule filler. The fluoxetine or placebo capsules are taken orally once daily for a total of 81 days; 60 days of treatment followed by a further 21 days whilst the first follow-up assessments are conducted to prevent confounding effects of washout at the 21-day test point owing to the short half-life of fluoxetine [69]. Participants will be asked to bring trial medication with them to each study visit where study researchers will perform pill counts for assessing adherence. Adherence will be measured by recording the number of pills returned and using this in conjunction with the number of doses supplied to calculate a percentage of the number of doses that should have been taken in the observed period. Although we will specifically be excluding participants with active depression, due to the risk of fluoxetine treatment increasing suicidal thoughts and suicidal ideation in adolescents [47–49], we will perform remote checks on the mental health on participants. This will be done via a telephone call with an experienced specialist epilepsy nurse 2 to 3 weeks after commencing treatment, when the risk is at its highest. Any participant indicating thoughts of suicide or depressive behaviour will be referred to the trial psychiatrist for immediate follow-up.

The dose chosen for the trial was on the basis of an equivalent dose being efficacious in animal models [31] and because it is below the limit at which tapering is required for medication withdrawal [70, 71], so participants will not receive tapered doses at the end of the treatment period or if cessation of trial treatment is required. If a participant who has stopped the trial treatment experiences any symptoms of withdrawal, they will be unblinded so that if they received fluoxetine, treatment can be resumed and gradually reduced over 1–2 weeks.

Participants will not be able to take any of the medicines listed in the exclusion criteria for the duration of the trial, but they will be permitted to continue with their current AED regime. Participants currently on carbamazepine therapy will be cautioned to self-monitor for exacerbation of drug side effects.

### Safety

Participants will be monitored for adverse events (AE) for the duration of the trial. Researchers will prompt participants to recount any adverse events experienced at each follow-up visit. Participants will also be encouraged to contact the team directly in between follow-up visits to report any incidence of AE to the trial team by the use of a trial-specific phone number monitored 24 h a day. Any AE that meets the criteria for a serious event will be reported immediately, and causality and expectedness will be assessed according to the Summary of Product Characteristics. We will forward standard timelines and procedures for the reporting of serious events to regulatory authorities.

### Feasibility outcomes

The primary outcome of this trial is feasibility which will be assessed by looking at measures of recruitment (number of participants recruited, percentage of people approached willing to participate and percentage of participants to pass screening), retention (percentage of participants still on study medication at follow-up assessment one and percentage of partially active participants at follow-up assessment one) and adherence (to study medication) according to pre-specified criteria, the detail of which can be found in Table 2. Each criterion is stratified into green (feasibility clearly demonstrated), amber (feasibility possibly demonstrated but requires further consideration) and red (feasibility not demonstrated). All criteria would need to be determined to be green or amber to progress to a full efficacy trial without major re-design of the study.

**Table 2:** Progression criteria for assessing feasibility of trial design

Variable		Progression criteria		
		Red	Amber	Green
Recruitment	Percentage of participants approached willing to participate	< 5%	5–20%	> 20%
	Percentage of participants screened actually recruited	< 50%	50–75%	> 75%
	Number of participants recruited	< 8	8–16	> 16
	Number of participants recruited within timeframe (1 year)	< 2	2–15	> 15
Retention	Percentage of participants still on study medication at follow-up assessment 1	< 30%	30–60%	> 60%
	Percentage of partially active* trial participants at follow-up assessment 1	< 50%	50–80%	> 80%
	Percentage of partially active trial participants at follow-up assessment 2	Not subject to progression criteria		
Adherence	Pill count (compliance) over 81 days	< 50%	50–80%	> 80%

*This would include participants that have partially withdrawn from the study, for example, stopping study medication but continuing with follow-up assessments

### Secondary outcome measures

The secondary outcome measures will include the participant’s performance in all cognitive assessments, performance in pattern separation task, hippocampal microstructure (as measured on 3T MRI) and seizure frequency. Learning and memory outcomes will be assessed across spatial, visual and verbal domains. The spatial domain will be assessed using the distance travelled and latency to find the hidden platform in the virtual water maze. Performance during probe trials will be measured using the proportion of time participants spend searching in the four quadrants of the pool and the platform area, the latency and distance travelled to cross the platform area, and the number of times the platform area was crossed. The visual domain will be assessed by using the recall accuracy of images recorded using the SenseCam wearable camera (scores), and the verbal domain will be assessed using word list learning and delayed recall scores.

For the pattern separation task, performance will be determined by the ratio of correct to incorrect responses and response latencies. Measures of hippocampal microstructure will be derived from diffusion-weighted MRI acquired before and after trial treatment. Seizure frequency will be determined from the analysis of events recorded in participant’s seizure diaries.

### Qualitative interviews

To gain a better understanding of factors influencing the participation of people with TLE in a trial such as this, we will be conducting semi-structured interviews with a variety of stakeholders. Interviews will be conducted with full written informed consent with (1) participants who agreed to take part in the trial, including those who were deemed to be ineligible; (2) participants who were invited to take part and declined; and (3) clinicians involved in the identification and recruitment of participants. We will aim to interview 15–20 people who have given written, informed consent to gain a wide range of views and opinions. Interviews will be conducted in a place of the participant’s choosing, which could include telephone interviews. Interviews will be audio recorded and transcribed verbatim for analysis.

### Data management and monitoring

All participants recruited to the trial will receive a unique participant identification number (PID) which will be used to anonymously identify all files and study materials relating to them. Personal identifiable data required for scheduling assessments will be stored in a password-protected database, separate from the main trial database on a securely networked computer and accessible only to the trial manager and CI.

For all standard cognitive assessments and validated rating scales, data will be collected in the appropriate published booklet and data entered into the on-line trial database. Questionnaire data will be collected via electronic case report forms (CRFs) allowing direct entry into the database. The computer-based cognitive assessment will automatically collect the required data, and the summary variables required for each test will be entered into the trial database. For all assessments, paper CRFs are available for recording data for circumstances where the trial database may be off-line. The trial database is held on securely networked servers with automatic backup. It is tested and validated with in-built range checks to ensure the accuracy and validity of data at the point of entry. All procedures for handling and storing both electronic and paper records are detailed in the trial data management plan.

Data will be monitored centrally on a routine basis and any queries raised directly with trial researchers. These will be monitored monthly by the trial management group. Trial steering (TSC) and data monitoring committees (DMC), independent of the trial sponsor, will be convened at regular intervals throughout the course of the trial. The DMC will review period safety data (unblinded if requested) and report any concerns to the TSC. If there are concerns that there is a clinically important difference in serious AE rates between study arms, then the TSC can make a decision to terminate the trial early with input from the DMC.

### Analysis

As this is a feasibility trial, no formal hypothesis testing will be conducted. The components of the primary outcome (recruitment, retention, adherence) will be presented as numbers and/or percentages, as appropriate, and assessed against pre-specified progression criteria (Table 2). All secondary outcomes will be summarised descriptively, by treatment arm. Categorical data will be summarised by number and percentage. Continuous data will be summarised by mean and standard deviation, or median and interquartile range if notably skewed. For each outcome, the relevant complete case population will be used, and participants will be analysed as randomised (intention-to-treat). Details of the analysis are specified in the trial statistical analysis plan.

For the qualitative interviews, transcribed interviews will undergo manual analysis to identify themes within the data using NVivo software. Identified themes will be used to categorise and summarise participant responses. A proportion of interviews (10%) will be randomly selected for double coding to ensure all relevant data is captured.

## Discussion

This feasibility trial is the first step towards determining the efficacy of fluoxetine as a treatment for the learning and memory problems associated with TLE [6]. The ultimate goal for the study is to see if a larger, fully powered efficacy trial investigating fluoxetine as a therapeutic agent for learning and memory problems in TLE is feasible and warranted.

This trial is designed to gather information on potential effect sizes in novel cognitive assessments translated from work in animal models of TLE and cognition. Further, we will be gathering information on the utility of relatively novel cognitive testing paradigms. Validation of such outcome measures could have wide-reaching implications for the study of cognition and in the investigation of novel therapies to improve learning and memory in epilepsy and other neurological disorders and represents a particular strength of this work. The investigation of fluoxetine in this context may also provide mechanistic insights into human adult cognition and the role of neurogenesis therein, albeit as an indirect measure as changes to human adult neurogenesis cannot be quantified. The purposeful exclusion of participants with active depression or anxiety has been incorporated into the study design to eliminate, as far as possible, the possible confounding effects of depression and anxiety on cognitive performance in the participants studied as depression status and cognitive performance are interlinked [72]. However, due to the high prevalence of depression and anxiety in people with epilepsy [73], this could have profound effects on recruitment rates and overall generalisability of this study, which is a limitation of the work.

The MR images captured as part of this study will be used to investigate whether fluoxetine treatment affects diffusion MRI measures of tissue microstructure in the hippocampus and/or its associated white matter pathways. To the best of our knowledge, this type of analysis has not been reported previously and is highly novel. Whilst this technique cannot directly measure any potential increase in neurogenesis, it will be interesting to see if fluoxetine treatment has any direct effect on hippocampal microstructure. We will also be able to investigate any structure-behaviour correlations between hippocampal microstructure metrics and performance in our cognitive tasks.

The findings of this trial will advance current knowledge in terms of understanding how fluoxetine is tolerated in people with epilepsy. The effect of fluoxetine on seizure frequency is still debated [35–39], so the tracking of seizure frequency in participants of this trial will provide further information for epilepsy clinicians concerned about prescribing fluoxetine to their patients.

A particular strength of this study will be realised through obtaining the views and opinions of participants taking part and perhaps, more importantly, those who chose not to take part in the trial will contribute to the understanding of potential barriers of facilitators underpinning the participation of people with epilepsy in this trial. Further, this information may also contribute to the understanding of why people with epilepsy may or may not take part in research and clinical trials in general, providing valuable information for the future development of research studies involving people with epilepsy.

Participants receiving the intervention in this trial have the potential to benefit from improved learning and memory using a relatively, cheap, widely available and generally well-tolerated medication, which may improve quality of life in people with TLE [8]. This could also have further societal benefits by reducing demand on carers and further enabling people with TLE to have active and productive working lives.

## Trial status

The trial is sponsored by Cardiff University (resgov@cardiff.ac.uk) and is currently open to recruitment. This manuscript was drafted according to version 8.0 (December 18, 2018) of the protocol. The protocol was written according to the Standard Protocol Items: Recommendations for Interventional Trials (SPIRIT) statement [74].

## Data Availability

This section is not applicable.

## Abbreviations

AED: Anti-epileptic drug
BMIPB: Brain Injury Rehabilitation Trust Memory and Information Processing Battery
BNF: British National Formulary
CI: Chief investigator
CRF: Case report form
DMC: Data monitoring committee
DT-MRI: Diffusion tensor magnetic resonance imaging
ECG: Electrocardiogram
EEG: Electroencephalogram
EMQ: Everyday memory questionnaire
FA: Fractional anisotropy
MD: Mean diffusivity
HADS: Hospital Anxiety and Depression Scale
HS: Hippocampal sclerosis
IQ: Intelligence quotient
3T MRI: 3 Tesla Magnetic Resonance Imaging
PICs: Participant identification centres
PID: Participant identification number
PIS: Participant information sheet
ROIs: Regions of interest
SPC: Summary of product characteristics
SSRI: Selective serotonin re-uptake inhibitors
TLE: Temporal lobe epilepsy
TOPF: Test of premorbid functioning
TSC: Trial steering committee
QOLIE-31: Quality of Life in Epilepsy-31
WASI: Wechsler Abbreviated Scale of Intelligence
